# Corneal Toxicity due to *Datura Inoxia*

**DOI:** 10.18502/jovr.v14i3.4792

**Published:** 2019-07-18

**Authors:** Rajesh Subhash Joshi

**Affiliations:** Department of Ophthalmology, Shri Vasantrao Naik Government Medical College, Yavatmal, India

**Keywords:** Datura, *Datura Inoxia*, Toxic Keratitis

## Abstract

**Purpose:**

To report corneal toxicity following intentional inoculation of the juice of crushed leaves of *datura* (*Datura Inoxia*).

**Case Report:**

A 70-year-old male presented with diminished vision, redness, watering, and photophobia in his right eye one day before his presentation. The patient had instilled the juice of *datura* leaves in his right eye to treat his ocular problems. Slit lamp examination revealed mild conjunctival and circumcorneal congestion, corneal edema, and folds in Descemet's membrane. The left eye was pseudophakic with an otherwise unremarkable examination. The patient was treated with dexamethasone, cycloplegics, and lubricants. The cornea did not sufficiently recover after one month of treatment leaving him with permanent corneal decompensation that required a referral for keratoplasty. The patient was followed up for six months. We hypothesize damage to the corneal endothelial Na+/K+-ATPase pump by tropane alkaloids as a cause for corneal decompensation.

**Conclusion:**

Awareness about toxicity of this commonly grown plant in the tropics and subtropics is essential in order to avoid blindness due to accidental or deliberate use.

##  INTRODUCTION

**Figure 1:**
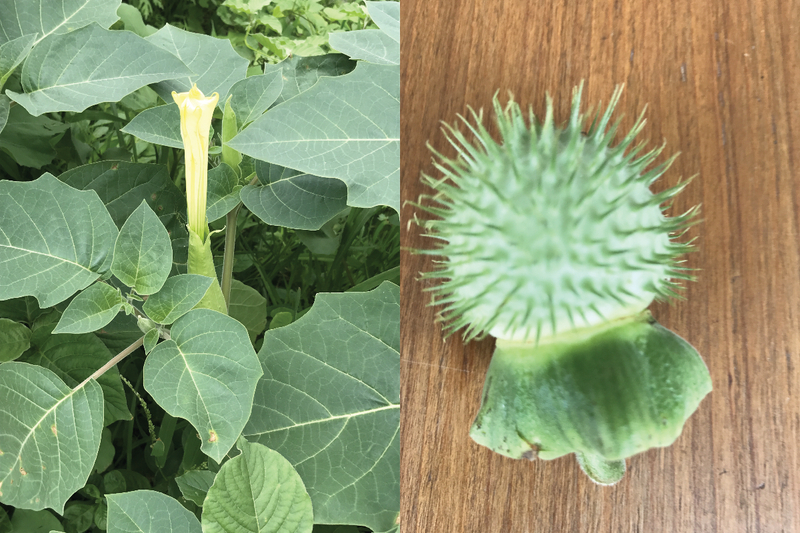
The leaves, flower and fruit of *datura inoxia*.

*Datura* belongs to a group of plants from the Solanaceae variety. They are commonly known as daturas or the devil's trumpets or moonflower.^[1]^ It is a popular ornamental plant in Western Europe.^[2]^ They are short-lived and can reach up to 2 meters in height. The leaves are alternately placed with a lobed or toothed margin. The flowers are erect or spreading trumpet-shaped. The color of the flower varies from white to yellow, pink, and pale purple. The fruit is a spiny capsule that releases numerous seeds when split open [Figure 1].

For centuries, *datura* has been used as a herbal medicine to relieve symptoms of asthma and as an analgesic during surgery. Other medicinal uses include relief from sore throat, relief from toothaches, antispasmodic medicine, antimalarial drug, treatment for patchy baldness, and antiparasitic medication.

*Datura* is found in the tropical and warm-temperate zones of wide areas of Asia, Africa, the Middle East, North America, Central America, and South America.^[3]^ In India, the *datura* flowers and leaves are used to worship Lord Shiva and Ganesh. Thus, it is not uncommon to get ocular injuries caused by accidental contact with the latex of leaves, flowers, or seeds of *datura* in rural or urban population. However, to the best of our knowledge, there are no reports of *datura* corneal toxicity. In this study, we report corneal toxicity caused by intentional ocular exposure to the leaves of the *datura* plant.

##  CASE REPORT

**Figure 2:**
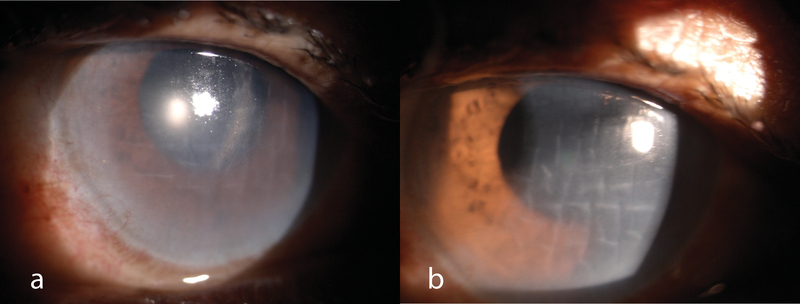
(a) Slit lamp photograph of a patient after exposure to the juice of *datura* leaves showing corneal edema and folds in the Descemet's membrane. (b) Slit lamp photograph showing persistent folds in Descemet's membrane at six months of follow up.

A 70-year-old man presented with a history of diminished vision, pain, redness, watering, and photophobia after instillation of juice of *datura* in his right eye. Based on his friend's advice, the patient had used the juice of *datura* leaves in his right eye to treat his ocular problems, one day before his presentation. The patient had foreign body sensation for the past two days in his right eye before the instillation of *datura* juice. He had undergone cataract surgery with implantation of intraocular lens three years earlier in his right eye and two years before in the left eye. The review of previous records revealed that his postoperative follow-up was uneventful. Visual acuity of the right eye was light perception. Slit lamp examination was not possible until the instillation of 0.5% proparacaine hydrochloride drops that allowed the patient to open his eye. The patient had a narrow palpebral aperture with conjunctival and circumcorneal congestion. Epithelial and stromal corneal edema was present. There were folds in Descemet's membrane [Figure 2(a)]. The pupil was dilated and did not react to direct light. No reaction was noted in the anterior chamber. The posterior chamber intraocular lens was in place. Corneal staining with fluorescein was negative. On ophthalmoscopy, a faint glow was visible, but nothing more specific could be noted. Optic disc and retinal vessels were not evaluable. The visual acuity in the left eye was 20/20 with a normal cornea and 3-mm pupil reacting to light. His systemic examination revealed tachycardia (pulse rate 100/minute). The rest of the systemic examination was normal.

The patient was medicated with 0.5% moxifloxacin eye drops four times a day, topical lubricants (Carboxymethylcellulose 0.5%, Refresh tear eye drop, Allergan India Pvt. Ltd.) four times a day, cycloplegics (Homatropine 2%, Homide, Indico Remedies Limited, India) three times a day, and dexamethasone 0.1% eye drops (Decolite, Intas Pharmaceuticals Pvt. Ltd. India) six times a day. Systemic non-steroidal anti-inflammatory and analgesic agents (Ibuprofen 400 mg and Paracetamol 325 mg, Combiflam, Sanofi India, Pvt. Ltd.)) were given for pain relief. On day 3, the patient experienced symptom relief. Visual acuity improved to 20/200. However, slit lamp examination still showed stromal edema and folds in Descemet's membrane. Intraocular pressure by applanation tonometry was 10 mm Hg in both eyes. Pulse rate was 80 beats/minute.

The patient was followed up for six months. No improvement in the visual acuity was noted. Corneal epithelial and stromal edema was still present. However, the epithelium was intact [Figure 2(b)]. Right eye fundus at six months was faintly visible with a normal appearing disc and retinal vessels. Foveal reflex could not be seen due to corneal edema. Due to the non-availability of specular microscopy, endothelial cell count could not be performed. Intraocular pressure was 10 mm Hg at six months of follow-up. The patient was referred for penetrating keratoplasty. The corneal graft was clear and there were no signs of recurrence at five months of follow-up.

##  DISCUSSION

*Datura* plants contain tropane alkaloids such as scopolamine, hyoscyamine, and atropine. These alkaloids have both medicinal and hallucinogenic properties. Tropane alkaloids are known to be absorbed through the corneal layers.^[4]^ Tropane has an action on the circular pupillary sphincter muscle, causing cycloplegia and mydriasis. There are reports of dilated fixed pupil after contact with the *datura* leaf.^[5,6]^ Self-limited mydriasis has been reported after simple exposure to the flowers of devil trumpet.^[7]^ However, corneal endothelial toxicity due to exposure to the leaf of *datura* has not been reported.

The patient was pseudophakic and previously had good vision in his right eye, which was documented on his previous records. The left eye was also pseudophakic and normal in all aspects. He had complained of foreign body sensation in the right eye for which he was told to instill the juice of crushed *datura* leaves by his close friend. He did not instill *datura* juice in his left eye.

The patient's presentation was similar to a corneal ulcer with the exception of no epithelial defect being present. Thus, dexamethasone eye drops were administered to reduce ocular inflammation. A study has demonstrated that steroids result in an increase in Na+/K+-ATPase pump activity in cultured corneal endothelial cells.^[8]^ Local application of steroids may increase pump activity in the remaining healthy endothelial cells leading to the recovery of cornea. However, in the present case, cornea did not recover and visual acuity did not return to the baseline. This could be due to the frequent instillation of *datura* juice in the present case. Basak et al presented a case series on ocular toxicity by latex of *Calotropis procera* and showed that the majority of eyes (17/24 eyes) had corneal endothelial loss in a follow-up period of three months in comparison to the fellow eyes.^[9]^ The epithelium remained intact. In our case, the epithelium was also found to be intact, but there were folds in Descemet's membrane and persistent corneal edema. This suggests that toxins liberated from the *datura* leaf are toxic to the endothelium without having any effect on the epithelium. This may be dependent on the dose of the juice instilled and the exposure to the cornea. Due to the non-availability of specular microscope, endothelial cell density could not be measured. Further studies are needed to determine the exact mechanism by which the toxins damage the endothelium while sparing epithelium. However, we hypothesize tropane alkaloids have their effect on Na+/K+-ATPase pump, leading to persistent corneal edema and folds in Descemet's membrane. Corneal endothelial Na+/k+-ATPase pump dysfunction affects the endothelial ion transport.^[10]^ This further causes loss of transparency and corneal edema and impairs vision. The specific concentration and duration of exposure of alkaloids that leads to the damage to the corneal endothelium is unknown and merits further research.

Toxic keratitis generally responds well to topical steroids, although some endothelial cell loss is natural in toxic endotheliitis. In this case, the corneal edema failed to resolve even at six months. This is a somewhat atypical sequelae of toxic keratitis/endotheliitis. Thus, it can be concluded that the juice of *datura* possibly causes corneal blindness. *Datura* has some historical and medicinal uses; therefore, people might use its extract to treat common ocular diseases. Therefore, public education is important to prevent accidental or intentional exposure to this widely distributed plant in Asian and Western countries to prevent its serious consequences on the cornea. Simple health tips like the use of gloves, washing hands, and avoiding the rubbing of eyes while plucking flowers, leaves, or seeds of *datura* may prevent such type of corneal blindness.

##  Financial Support and Sponsorship

Nil.

##  Conflicts of Interest

There are no conflict of interests.
